# Supplementary material for: Multifunctional Properties of a Bacillus thuringiensis Strain (BST-122): Beyond the Parasporal Crystal
Source: Toxins (Basel). 2022 Nov 7;14(11):768. doi: 10.3390/toxins14110768 (PMC9695252; doi:10.3390/toxins14110768)
Supplement: Supplementary file 1 [file toxins-14-00768-s001.zip › toxins-1991597-supplementary.pdf]

toxins

MDPI

Article

Multifunctional Properties of a *Bacillus thuringiensis* Strain (BST-122): Beyond the Parasporal Crystal

Argine Unzue, Carlos J. Caballero, Maite Villanueva, Ana Beatriz Fernández and Primitivo Caballero

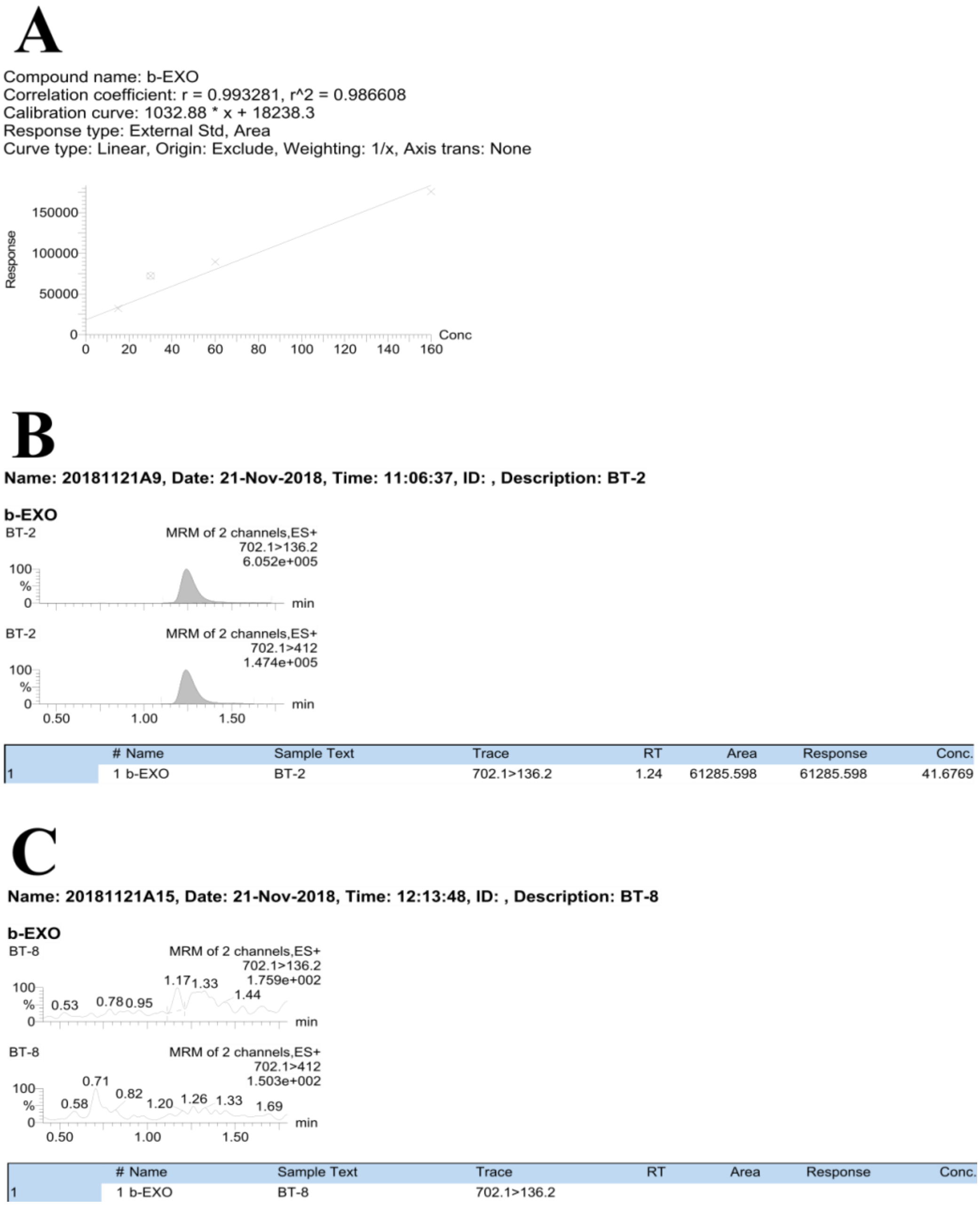

Figure S1. Results of HPLC analysis for detection of production of type I β-exotoxin (thuringiensin). (**A**): Calibration curve of HPLC analysis, * multiplication symbol; (**B**): HD-2 strain standard results (positive control); (**C**): BST-122 results.

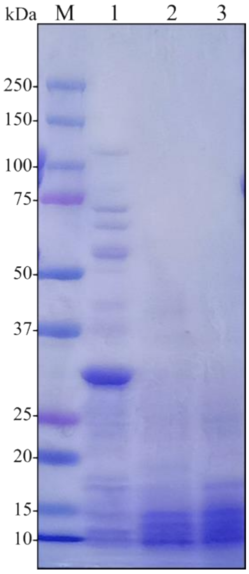

Figure S2. SDS-PAGE from BST-122 strain. M: protein weight marker (kDa); 1: spores and crystal mixture; 2: solubilized in carbonate buffer (pH 11.3); 3: solubilized protein dialyzed in HEPES (pH 8.0).

Table S1. Mean lethal concentration (LC_{50}) value of the BST-122 solubilized protein crystals for J2 *M. incognita* individuals.

LC	Concentration (μg/ml)	Lower limits	Upper limits	χ^2	df	Slope	SE slope	Intercept
LC_{50}	458	276	11711	24.0	6	1.37	0.224	−3.64

LC: lethal concentration; χ^2: chi-square; df: degree of freedom; SE: standard error.

Table S2. Mortality percentages of J2 *M. incognita* individuals treated with Cry5_orf65 solubilized protein crystals.

Treatment	Concentration (μg/ml)	Mortality (%) Mean ± SE
Control (HEPES)	0	14.5 ± 5.2 a
BMB171-pSTAB-empty	50	37.7 ± 2.9 a
	150	97.1 ± 2.9 b
BMB171-Cry5_orf65	50	50.7 ± 20.4 ab
	150	86.9 ± 2.5 b

SE: Standard Error. Different letters were used to denote statistical significance between values. Kruskal-Wallis (χ^2 = 11.552, df = 4, p = 0.02101), Bonferroni test (p-value <0.05).

Table S3. Insecticidal protein content of *Bacillus thuringiensis* BMB171.

Target Database[a]	Identity (%)[b]	MW (kDa)	Length (No. Residues)
CalY	55	22.1	197
**Endochitinase**	99	75.8	688
**Bmp1**	35	60.9	566
InhA2	99	87.9	799
Sphingomyelin phosphodiesterase	99	33	294
DNRLRE domain-containing protein	100	251.7	2253
CalY	62	21.8	197
CalY	99	20.7	189

InhA1	96	86.7	796
InhA2	29	72.9	656
**Bmp1**	97	89.4	817
InhA3	99	76.2	692
InhA1	78	86.9	795
**Bmp1**	61	62.4	567
Hemolytic enterotoxin HBL	99	52.3	466
**Bmp1**	61	62.5	565
Enhancin_Bel	100	85.6	742
**Exochitinase**	99	39.4	360
**ColB**	78	48.3	426
**ColB**	34	35.6	309
CalY	50	22.3	201
**Bmp1**	36	65.3	591
Spp1Aa1	82	40.2	362
**Bmp1**	32	64.6	583

[a]**Bold**, described toxicity to nematodes. [b]Local alignment using BLASTP.

Table S4. Comparative of strains BST-122 and BMB171 secreted factors.

**Target Database**[a]	**BST-122**		**BMB171**	
	**% Identity**[b]	**MW (kDa)**	**% Identity**[b]	**MW (kDa)**
**Bmp1**	36	65.3	97	89.4
**ColB**	78	48.3	78	48.3
**Endochitinase**	-	-	99	75.8
**Exochitinase**	99	39.4	99	39.4
CalY	56	22	99	20.7
InhA1	96	86.7	96	86.7
InhA2	99	87.9	99	87.9
InhA3	-	-	99	76.2
Enhancin_Bel	99	85.5	100	85.6
Sphingomyelin phosphodiesterase	100	37.0	99	33.0

[a]**Bold**, described toxicity to nematodes. [b]Local alignment using BLASTP.

Table S5. Primers used in this study.

**Primers**	**Sequences (5′-3′)**[a]
For gene cloning	
Cry5Ad-like_NcoI_FW	CC*CATGG*CCAAATGATTGCATATCTTGATT
Orf2_Cry65_Eco52I_RV	CC*CGGCCG*CCTCCGCGTGTATTCTTATA
For gene sequencing	
Cry5As-like-seq-FW	CATTACAGCAATTGCACACC
Cry5As-like-seq-RV	CCAGTAACAACAAAGCTGC

[a]*Italic*, restriction enzyme site included in the primer sequence.
